# Turning data into research-ready data

**DOI:** 10.23889/ijpds.v8i2.2217

**Published:** 2023-09-14

**Authors:** Van Phan, Felix Ritchie, Bryson Alex, John Forth, Lucy Stokes, Damian Whittard

**Affiliations:** 1 University of the West of England, Bristol, United Kingdom; 2 University College London, London, United Kingdom; 3 City university, London, United Kingdom; 4 NIESR, London, United Kingdom

## Objectives

Governments acquire extensive data holdings and face increasing pressure to make these available as record-level microdata for research. However, turning data into research-ready data (RRD) is not a straightforward exercise. We demonstrate how even in simple cases researcher involvement can bring substantial rewards for effective RRD development.

## Method

This paper reports on an ADRUK-funded project to take a dataset originally collected by the Office for National Statistics for official statistics (the UK Annual Survey of Hours and Earnings, ASHE), formally review its microanalytical characteristics, link it to Census 2011 data, and prepare a new ‘research ready dataset’ with appropriate documentation and coding. This should have been straightforward as the datasets had already been widely used as research microdata. However, the involvement of academic researchers in the production of research-ready data led to many important new insights.

## Results

The research programme had 3 aims: testing assumptions about the data; reviewing data quality; and adding value.

Because of its sampling model, ASHE is assumed to have random non-response both longitudinally and in cross section. The research team showed that was untrue: there was higher attrition than expected, and both longitudinal and cross-sectional non-response appeared non-random..

The data quality review showed further concerns about the accuracy of some geographical indicators, and some variables of opaque provenance; in contrast, we confirmed the accuracy of administrative variables created by ONS.

As well as being important for researchers, these findings have the potential for significant effects on official statistics produced from the source data, enhancing the value of the source data.

Finally, value was added from new variables which reflected the team’s wide research interests.

## Conclusion

Often in government the assumption is that creating RRDs is a matter of creatign files and giving access to the researchers. Insights from our work show that the deep involvement of the research community can bring rewards for both data holders and researchers. For RRDs, researcher-led construction is vital.

